# The relationship between the isocapnic buffering phase and maximal accumulated oxygen deficit in athletes

**DOI:** 10.3389/fphys.2026.1947289

**Published:** 2026-09-22

**Authors:** Muhammed Ay, Selcen Korkmaz Eryılmaz, Burcu Özdemir, Abdullah Kılcı, Ömer Cumhur Boyraz, Özgür Günaştı, Mustafa Oğuzhan Akbulut, Çiğdem Özdemir Postallı, Ertuğrul Gezgin, Erkan Tiyekli, Kerem Özgünen, Sanlı Sadi Kurdak

**Affiliations:** 1 Department of Coaching Education, Faculty of Sports Sciences, University of Esenyurt, Istanbul, Türkiye; 2 Department of Physical Education and Sports Teaching, Faculty of Sports Sciences, University of Cukurova, Adana, Türkiye; 3 Department of Coaching Education, Faculty of Sports Sciences, University of Cukurova, Adana, Türkiye; 4 Department of Physiology, Faculty of Medicine, University of Cukurova, Adana, Türkiye; 5 Faculty of Dentistry, University of Cukurova, Adana, Türkiye

**Keywords:** aerobic capacity, anaerobic capacity, cardiopulmonary exercise testing, oxygen uptake, respiratory compensation point, Wingate anaerobic test

## Abstract

**Purpose:**

This study aimed to investigate the relationship between the isocapnic buffering (IB) phase and maximal accumulated oxygen deficit (MAOD) in athletes.

**Methods:**

Twenty-two active male athletes (19.7 ± 1.4 years) participated in an incremental test to determine maximal oxygen uptake (V̇O_2max_), the intensity associated with V̇O_2max_ (vVO_2max_), the anaerobic threshold (AT), the respiratory compensation point (RCP), and IB, 6 submaximal efforts to construct the V̇O_2_-velocity relationship, a supramaximal effort to determine MAOD, and the Wingate Anaerobic test. The IB was calculated as the difference in both oxygen uptake (IB_VO2_) and running velocity (IBv) between the RCP and AT, and expressed in absolute or relative values.

**Results:**

MAOD was positively associated with absolute and relative IB_VO2_ and IB_V_ (rs=0.825, 0.753, 0.675, and 0.630, respectively; all Holm-adjusted p ≤ 0.002). In exploratory analyses, peak power remained associated after false discovery rate (FDR) adjustment with absolute and relative IB_VO2_, IB_V_, and MAOD (q ≤ 0.047), while mean power remained associated with absolute and relative IB_VO2_ and MAOD (q ≤ 0.047). No statistically significant association was detected between V̇O_2max_ and either MAOD or the IB phase.

**Conclusion:**

These findings suggest that the IB phase may be a potential physiological indicator associated with anaerobic capacity in athletes. A high buffering capacity may allow anaerobic glycolysis to persist longer, thereby providing greater anaerobic capacity.

## Introduction

Exercise physiologists, coaches, and athletes need access to information on the characteristics that contribute to athletic performance to design and implement effective training programs. Various aerobic and anaerobic test parameters are available to assist in planning and monitoring training programs. Among these parameters, maximal oxygen uptake (V̇O_2max_) and anaerobic threshold (AT) are important physiological variables used to assess endurance performance capacity (Hasanli et al., 2015). On the other hand, anaerobic performance capacity is commonly evaluated with the Wingate anaerobic test (Bar-Or, 1987) and maximal accumulated oxygen deficit (MAOD) (Medbo et al., 1988). Additionally, the isocapnic buffering (IB) phase is considered to provide indirect predictive information about the buffering capacity of metabolic acidosis during exercise (Whipp et al., 1989). However, the relationship of the IB phase to aerobic or anaerobic capacity has not been fully elucidated.

The maximum aerobic and anaerobic energy production capacities are critical in determining or limiting athletic performance across different sports (Rajpoot et al., 2025; Dubey and Choudhary, 2023). In contrast to aerobic metabolism, it is difficult to measure the contribution of oxygen-independent anaerobic metabolic pathways (glycolytic and phosphagen) to total energy production during exercise (Gastin, 1994; Medbo et al., 1988). Since direct muscle tissue sampling is not always feasible, MAOD is commonly used as an indirect method to estimate anaerobic capacity (Medbo et al., 1988; Noordhof et al., 2010). The MAOD calculation, owing to its validity and reproducibility, relies on subtracting the aerobic contribution from the total energy production during supramaximal exercise to assess anaerobic exercise capacity (Medbo et al., 1988; Noordhof et al., 2010; Weber and Schneider, 2001; Gastin, 1994). The significant correlations between MAOD and blood and muscle lactate, creatine phosphate, and ATP levels indicate that the greater the MAOD value for a given exercise period, the greater the contribution of anaerobic ATP production (Medbo et al., 1988; Bangsbo et al., 1990; Medbo and Tabata, 1993; Tanji et al., 2017). The MAOD is used to evaluate training status (Zagatto et al., 2017), determine the metabolic profile of high-intensity exercise (Spencer and Gastin, 2001), and validate anaerobic tests (Scott et al., 1991; Maxwell and Nimmo, 1996).

During an incremental exercise test, the AT (metabolic or gas-exchange threshold) and the respiratory compensation point (RCP) are typically defined as two separate thresholds used to assess performance capacity (Wasserman et al., 2012). AT corresponds to the exercise intensity at which the contribution of anaerobic metabolism to overall metabolism begins to increase, and reflects the onset of metabolic acidosis (Wasserman et al., 2012; Beaver et al., 1986). At exercise intensities just above the AT, excess (non-oxidative) carbon dioxide (CO_2_) is released as bicarbonate buffers the increased H^+^, leading to a disproportionate increase in V̇CO_2_ relative to the increase in V̇O_2_ (Beaver et al., 1986; Wasserman et al., 2012). Ventilation (VE) increases proportionally with V̇CO_2_ to maintain arterial partial CO_2_ pressure and regulate pH (Beaver et al., 1986; Wasserman et al., 2012; Binder et al., 2008). As exercise intensity increases, the buffering systems eventually reach a saturation point, and further stimulation of the chemoreceptors causes hyperventilation, which corresponds to RCP (Wasserman et al., 2012; Meyer et al., 2004; Whipp et al., 1989; Binder et al., 2008). The period between AT and RCP is defined as the IB phase, representing the compensatory phase of exercise-induced metabolic acidosis (Wasserman et al., 2012; Whipp et al., 1989; Takano, 2000). Due to the balance between lactate and H^+^ production and buffering systems, athletes may sustain exercise for a long duration at intensities within the IB range. The phase from RCP to the end of the exercise is referred to as hypocapnic hyperventilation (HHV) (Chicharro et al., 2000; Oshima et al., 1998).

High buffering capacity may enable anaerobic glycolysis to be sustained for extended periods without reaching performance-limiting pH levels (Nakagawa and Hattori, 2002; Edge et al., 2006). Thus, the ability to buffer H^+^ is likely associated with anaerobic capacity (Hasanli et al., 2015). The IB phase may differ among athletes due to variations in adaptive variables such as lactate kinetics, buffering capacity, and carotid body sensitivity to metabolic acidosis (Whipp et al., 1989; Takano, 2000), depending on their training regimens (Hasanli et al., 2015; Hirakoba and Yunoki, 2002; Edge et al., 2006). Potential shifts in the AT and RCP due to physical training may affect the IB phase (Chicharro et al., 2000; Oshima et al., 1998; Erylmaz et al., 2018). Assessing the IB phase can be useful for coaches and physiologists to characterize the metabolic adaptations triggered by a specific training regimen (Hasanli et al., 2015).

Previous studies have shown inconsistent results regarding the use of the IB phase to assess aerobic or anaerobic components. Studies on the IB phase have mostly focused on its relationship to aerobic rather than anaerobic capacity (Oshima et al., 1998; Lenti et al., 2011; Yen et al., 2018; Korkmaz Eryılmaz et al., 2018). Only one study has examined the relationship between MAOD and the IB phase by evaluating the changes in blood lactate (Hasanli et al., 2015). However, to our knowledge, the IB phase calculated from gas exchange data and the MAOD relationship has not been investigated previously. With this in mind, this study aimed to investigate the relationship between the IB phase and MAOD in athletes.

## Materials and methods

### Participants

Twenty-two male athletes (19.7 ± 1.4 years, 179.2 ± 7.2 cm, and 73.8 ± 9.1 kg) from various sports, including 10 soccer players, 4 basketball players, 4 sprinters, and 4 field hockey players, volunteered for the study. The inclusion criteria for this study were no health problems, being active in running-based sports, and training for at least 6 hours per week. Participants who smoked, had a history of any disease, used any drugs or ergogenic aids that affected physiological responses, or had an irregular training status were excluded from the study. To account for potential participant loss, we initially included 25 participants. However, we ultimately analyzed data from 22 participants because two did not complete their lab visits, and one participant’s data were unsuitable for analysis due to irregular ventilation. All participants had trained and competed regularly for at least 5 years (6.9 ± 1.8 years). Participants completed a health history questionnaire, were free of chronic health problems, had no contraindications to maximal exercise testing, and had no musculoskeletal injury in the past six months. The test procedures used in this study were approved by the Ethics Committee of Çukurova University Faculty of Medicine, in accordance with the Declaration of Helsinki (149/57/2024).

### Experimental design

All participants attended at least 5 visits to the Çukurova University Healthy Life and Sports Sciences Research and Application Center Laboratory, which were separated by 24–72 h. They were instructed to maintain their usual dietary habits, eat a light meal about 2 h before testing sessions, stay hydrated, maintain normal sleep patterns, attend each session under similar daily conditions, and avoid additional strenuous physical activity, alcohol, and caffeine during the experimental period. We obtained verbal confirmation of compliance before each testing session. The participants completed all testing at the same time of day (± 1 h) to eliminate any possible influence of circadian variations. The participants were familiarized with the experimental procedures and equipment, and written informed consent was obtained before testing.

During the first visit, maximal incremental exercise tests were performed to determine V̇O_2max_, the intensity associated with V̇O_2max_ (vV̇O_2max_), AT, and RCP. Participants underwent a total of six 8-minute submaximal efforts at a constant velocity between 40% and 90% vV̇O_2max_ to construct V̇O_2_-velocity relationships in the following two sessions. In the fourth session, a supramaximal effort (115.1 ± 3.7% of vV̇O_2max_) was performed to determine accumulated oxygen uptake, accumulated oxygen demand, and MAOD. In the final session, participants performed the Wingate Anaerobic test to evaluate anaerobic power and capacity. To standardize results by reducing fatigue effects, participants completed the tests in the same order. The tests were completed within two weeks of each other, between the first and last visits. At least 48 h were provided between visits. However, at the request of some participants who were feeling well, a 24 h interval was provided between the second and third visits, which were two submaximal testing sessions. Tests that did not meet the criteria were repeated: the supramaximal effort test for five participants and the maximal incremental exercise test for two participants, with at least a 48 h interval between test sessions.

Before each test, participants completed a 10-minute warm-up on a treadmill or bicycle at their preferred intensity, followed by approximately 5 minutes of stretching. Following the warm-up and stretching section, participants rested for ten minutes to return to their resting metabolic state. All participants were verbally encouraged to exert maximal effort during the incremental, supramaximal, and Wingate tests. The incremental, submaximal, and supramaximal effort tests were performed on a motorized treadmill (HP Cosmos Nussdorf-Traunstein, Germany) at a fixed 1% incline. Pulmonary gas exchange was measured breath-by-breath throughout the exercise using a metabolic gas analysis system (PFT Cosmed, Rome, Italy) calibrated before each session. Heart rate (HR) was measured by telemetry using an HR monitor (Cosmed, Rome, Italy). To reduce short-term respiratory variability and measurement noise, breath-by-breath data were averaged in 10 s intervals using the time-averaging function of the Cosmed Omnia software. Subsequently, the averaged data were smoothed using an 8-point smoothing procedure to minimize residual fluctuations.

#### Incremental exercise test

The incremental exercise test began at 7 km/h, and treadmill velocity increased 1 km/h per minute until participants could no longer maintain the running pace. Tests were terminated by volitional exhaustion. The highest 10-second V̇O_2_ value over the last stage of the test was defined as the V̇O_2max_ by considering at least two criteria: plateau in V̇O_2_ (change < 2.1 ml/kg/min) despite increasing velocity; respiratory exchange ratio (RER) ≥1.10; and maximal heart rate (HR_max_) ≥ 90% of predicted for age (220 – age). The incremental test V̇O_2max_ values were validated with supramaximal testing on the fourth test day. Two participants did not meet the criteria for achieving V̇O_2max_, so the incremental exercise test was repeated on a different day. vV̇O_2max_ was defined as the lowest running velocity at which V̇O_2max_ was achieved. If the final stage of the incremental exercise test had not been completed, the running velocity of the previous stage was recorded as vV̇O_2max_. Time to exhaustion (min) was recorded as the duration from the beginning of the incremental test until the participant could no longer maintain the running velocity.

### Determination of the anaerobic threshold and the respiratory compensation point

The V-slope method was utilized to determine AT and RCP (Beaver et al., 1986; Wasserman et al., 2012). AT and RCP were defined as the intersection point of the two linear regression lines, indicating disproportionate increases in the V̇CO_2_ versus V̇O_2_ and VE versus V̇CO_2_ relationships, respectively. Additionally, a visual identification technique was used to improve the accuracy of AT and RCP identification, as previously published (Korkmaz Eryılmaz et al., 2024). The AT was determined using the criterion of increases in the ventilatory equivalent for oxygen (VE/VO_2_) and end-tidal oxygen pressure (PETO_2_) without simultaneous changes in the ventilatory equivalent for carbon dioxide (VE/V̇CO_2_) and end-tidal carbon dioxide pressure (PETCO_2_), while the RCP corresponded to an increase in the VE/V̇CO_2_ and a decrease in the PETCO_2_ (Wasserman et al., 2012; Binder et al., 2008). Two independent investigators performed blinded data analysis. Both investigators analyzed all the data twice in a random order, allowing at least one week between assessments, and reached inter-assessment consensus. The difference between investigators was no more than 30 s (i.e., three data points), and the average value was used for data analysis. If a disagreement involves more than three data points, the opinion of a third investigator will be sought; however, no disagreement arose. The intraclass correlation coefficient (ICC[2,1]) between the two investigators was 0.999 (95% CI: 0.997–0.999; p < 0.001) for AT and 0.997 (95% CI: 0.992–0.999; p < 0.001) for RCP. The V̇O_2_ and running velocity at the AT (AT_VO2%_ and AT_V_ %, respectively) and at the RCP (RCP_VO2%_ and RCP_V_ %, respectively) were presented as percentages of V̇O_2max_ and vV̇O_2max_, respectively.

### Determination of the isocapnic buffering phase

The IB phase was calculated as the difference in both V̇O_2_ (IB_VO2_) and running velocity (IBv) between the RCP and AT (Oshima et al., 1998). Additionally, the IB phase was expressed as a relative % of the RCP as previously described by Röcker et al. (1994). The HHV was calculated as the difference in V̇O_2_ (HHV_VO2_) between V̇O_2max_ and RCP and was expressed both as an absolute value and as a relative % of V̇O_2max_ (Oshima et al., 1998; Lenti et al., 2011). Additionally, the running velocity (HHVv) difference between vV̇O_2max_ and RCP was expressed in both absolute and relative % of vV̇O_2max_.

#### Submaximal tests

To minimize the potential effect of fatigue, submaximal tests were conducted sequentially from low to high intensity. Participants completed the three submaximal efforts at 40%, 60%, and 80% of vV̇O_2max_ in the second test session. They were invited to perform the three submaximal efforts at 50%, 70%, and 90% of vV̇O_2max_ in the third test session. Between submaximal efforts, the subjects rested in a sitting position for 15 minutes to allow V̇O_2_ to return to baseline. The V̇O_2_ during the last 2 minutes of each submaximal effort was averaged to obtain the steady-state V̇O_2_ for the corresponding velocity, which was used to construct the V̇O_2_-velocity relationship.

#### Supramaximal test

Following the warm-up period, participants completed two 15-s high-velocity treadmill trials at 80% and 100% of their supramaximal velocity, with a 2-minute recovery between trials to familiarize them with the test velocities. Supramaximal exercise intensity for each participant was determined individually to fully exhaust them within approximately 2 minutes at 115% of vV̇O_2max_, based on several short trials at their self-selected speed and previous tests. Participants were instructed before the test and encouraged throughout to exercise for as long as possible, but they were unaware of the elapsed time. If the target duration was exceeded by more than ± 30 s, the test was repeated on a separate day with the exercise intensity recalculated. Five participants had their supramaximal test repeated because they exceeded the target time. The average resultant velocities for the supramaximal test were 19.5 ± 1.08 km/h at 115.1 ± 3.7% of vV̇O_2max_ for 2.06 ± 0.2 minutes.

### Determination of the maximal accumulated oxygen deficit

For each participant, a linear regression line was fitted to six submaximal velocities and their respective steady-state V̇O_2_ values, then extrapolated to estimate oxygen demand for the supramaximal test velocity (**Figure 1A**). The linear equation was considered valid only if the r² value exceeded 0.99 (r² = 0.994 ± 0.002). During the supramaximal test, V̇O_2_ data were analyzed in 10-s periods (**Figure 1B**). Oxygen demand was assumed to be constant throughout supramaximal exercise. MAOD was calculated as the sum of the differences between the estimated oxygen demand area (accumulated oxygen demand) and the actual oxygen uptake area (accumulated oxygen uptake) for each 10-s period of the supramaximal test.

**Figure 1:**
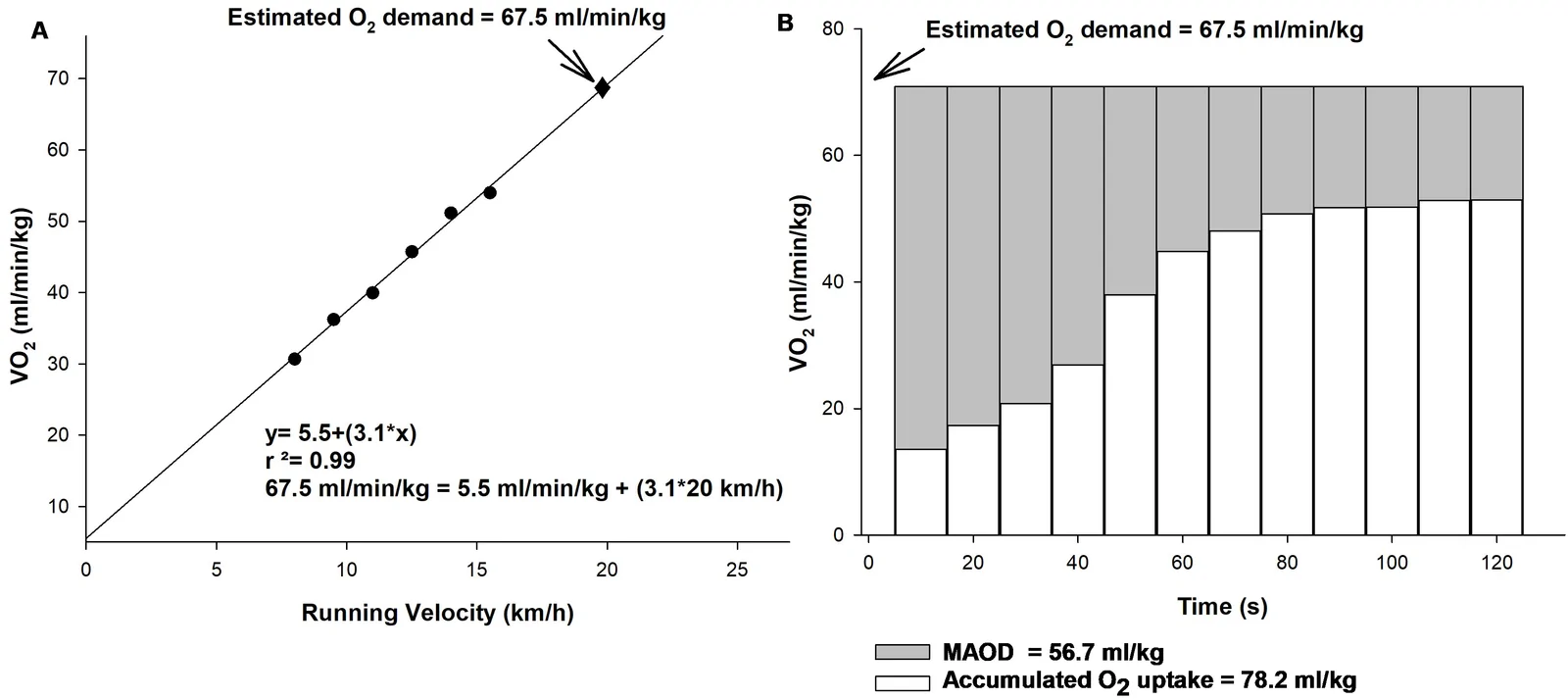
Illustration of the calculation of maximal accumulated oxygen deficit (MAOD) for a participant with a V̇O_2max_ of 53.9 ml/kg/min. The participant ran to exhaustion for 120 s at 117.6% vVO_2max_ (20 km/h) during the supramaximal test. **(A)** A linear regression line was constructed based on the relationship between the V̇O_2_ responses and the running velocity obtained from six submaximal exercises. The estimated oxygen demand was then found to be 67.5 ml/min/kg by extrapolating this regression line up to a running velocity of 20 km/h. **(B)** During the 120-s to exhaustion, the accumulated oxygen demand (sum of white and gray areas) was 135 ml/kg. The accumulated oxygen uptake (white area) was 78.2 ml/kg, giving a MAOD (gray area) of 56.7 ml/kg.

### Wingate anaerobic test

The Wingate anaerobic test was conducted on a computerized cycle ergometer (834 E, Monark) in the standard version (Inbar et al., 1996). Participants performed a 5-minute warm-up on a cycle at 60–70 rpm, including two 5-s sprints at the end of the 3^rd^ and 5^th^ minutes, followed by about 5 minutes of stretching. Participants pedaled at maximal effort for 30-s against a resistance equivalent to 7.5% of their body mass, following a 3-s acceleration phase. Participants were instructed to pedal as fast as possible before the test and were verbally encouraged to maintain the highest possible pedaling rate throughout the 30-s. The peak power, mean power, and fatigue index were calculated using the Monark Anaerobic Test Software.

#### Statistical analyses

Data are reported as mean ± standard deviation (SD). Normality was assessed separately for each variable using the Shapiro-Wilk test and by visual inspection. MAOD, IBv, and HHVv were non-normally distributed (p=0.001, p=0.004, and p=0.005, respectively); the remaining analysis variables did not show evidence of non-normality (all p>0.05). Pearson’s product-moment correlation coefficient (r) was used when both variables were normally distributed, whereas Spearman’s rank correlation coefficient (rs) was used when at least one variable was non-normally distributed. Partial Spearman correlations were calculated by applying Pearson partial correlation to the ranked variables. The four relationships of MAOD with absolute and relative IB_VO2_ and IBv constituted the primary analysis family; Holm’s procedure controlled the family-wise error rate across these four tests. All correlations involving Wingate, HHV, and incremental-test variables, together with the partial correlations, were treated as secondary and exploratory. The Benjamini-Hochberg procedure controlled the false discovery rate across the 73 exploratory tests. Two-sided raw p values and multiplicity-adjusted p or q values are reported. IBM SPSS Statistics 21 (IBM SPSS Inc., Chicago, IL) was used for the original data management, and the revised correlation analyses were independently verified using Python 3.12 with SciPy 1.17. Analyses for determining MAOD, AT, and RCP were performed using Sigma Plot 12.0 (Systat Software Inc., Chicago, USA).

No formal *a priori* sample-size calculation was conducted. The study included 22 male athletes who met the eligibility criteria and completed the study procedures. A sensitivity analysis indicated that, with n=22, a two-sided α level of 0.05 and 80% power, the minimum detectable correlation was approximately |r|=0.55. Thus, the available sample was primarily capable of detecting relatively strong correlations. For Pearson correlations, 95% confidence intervals were calculated using Fisher’s r-to-z transformation. For Spearman correlations, bias-corrected and accelerated bootstrap 95% confidence intervals were estimated using 5,000 paired resamples.

Four *post hoc* ordinary least-squares regression models were performed as sensitivity analyses, with MAOD as the outcome. Because IB is defined deterministically as RCP minus AT, simultaneous inclusion of AT, RCP, and IB would produce a rank-deficient design matrix and perfect structural multicollinearity. Therefore, each model included the corresponding AT position and IB measure: absolute AT_VO2_ and IB_VO2_; AT_VO2_% and relative IB_VO2_; absolute ATv and IBv; or ATv% and relative IBv. For the absolute models, the AT-plus-IB parameterization is algebraically equivalent to an AT-plus-RCP model. The IB coefficient estimates its association with MAOD at a fixed AT position; it should not be interpreted as demonstrating biological or causal independence. Variance inflation factors (VIFs), Shapiro-Wilk tests of residuals, Breusch-Pagan tests, leverage, and Cook’s distance were examined. Given the small sample, coefficient inference used HC3 heteroscedasticity-consistent standard errors. Benjamini-Hochberg FDR adjustment was applied across the four IB coefficients. Leave-one-out analyses were used to assess sensitivity to individual observations.

## Results

The results from the incremental test, supramaximal test, and Wingate anaerobic test are shown in **Table 1**. All four primary MAOD-IB associations were positive and remained statistically significant after Holm adjustment. MAOD was associated with absolute IB_VO2_ (rs=0.825, bootstrap 95% CI: 0.538-0.935; Holm-adjusted p<0.001), relative IB_VO2_ (rs=0.753, 95% CI: 0.426-0.901; Holm-adjusted p<0.001), IBv (rs=0.675, 95% CI: 0.276-0.863; Holm-adjusted p=0.001), and relative IBv (rs=0.630, 95% CI: 0.201-0.836; Holm-adjusted p=0.002) (**Figures 2A–D**).

**Table 1:** Variables from the incremental exercise test, supramaximal effort test, Wingate anaerobic test, isocapnic buffering (IB), and hypocapnic hyperventilation (HHV) phases (Mean ± SD).

Incremental exercise test variables		IB and HHV phase variables	
V̇O_2max_ (ml/kg/min)	50.2 ± 3.7	IB_VO2_ (ml/min/kg)	10.2 ± 2.2
HR_max_ (beat/min)	195.2 ± 8.3	Relative IB_VO2_ (%)	21.6 ± 4.4
Time to exhaustion (min)	10.6 ± 1.2	IB_V_ (km/h)	4.3 ± 0.9
vVO_2max_ (km/h)	17.1 ± 1.2	Relative IB_V_ (%)	29.3 ± 5.8
VE_max_ (L/min)	151.5 ± 21.6	HHV_VO2_ (ml/min/kg)	2.8 ± 1.8
RER_max_	1.2 ± 0.06	Relative HHV_VO2_ (%)	5.6 ± 3.7
V̇O_2_ at the AT (ml/kg/min)	37.1 ± 4.01	HHV_V_ (km/h)	2.2 ± 0.7
V̇O_2_ at the RCP (ml/kg/min)	47.3 ± 4.07	Relative HHV_V_ (%)	13.2 ± 4.3
AT_VO2%_ of V̇O_2max_	73.8 ± 5.1	Supramaximal effort test variables	
RCP_VO2%_ of V̇O_2max_	94.3 ± 3.7	MAOD (ml/kg)	58.8 ± 6
AT_V_ % of vV̇O_2max_	61.2 ± 4.9	AO_2_ demand (ml/kg)	136.2 ± 16.07
RCP_V_ % of vV̇O_2max_	86.7 ± 4.3	AO_2_ uptake (ml/kg)	77.4 ± 14.04
Wingate anaerobic test variables			
Peak power (W/kg)	10.3 ± 1.3		
Mean power (W/kg)	7.7 ± 0.8		
Fatigue index (%)	54.2 ± 6.8		

Max, maximal values of physiological variables; V̇O2, oxygen uptake; HR, heart rate; RER, respiratory exchange ratio; vV̇O2max, minimum velocity which elicits V̇O2max; VE, minute ventilation; AT, anaerobic threshold; RCP, respiratory compensation point; v, velocity; AO2, accumulated oxygen; MAOD, maximal accumulated oxygen deficit.

**Figure 2:**
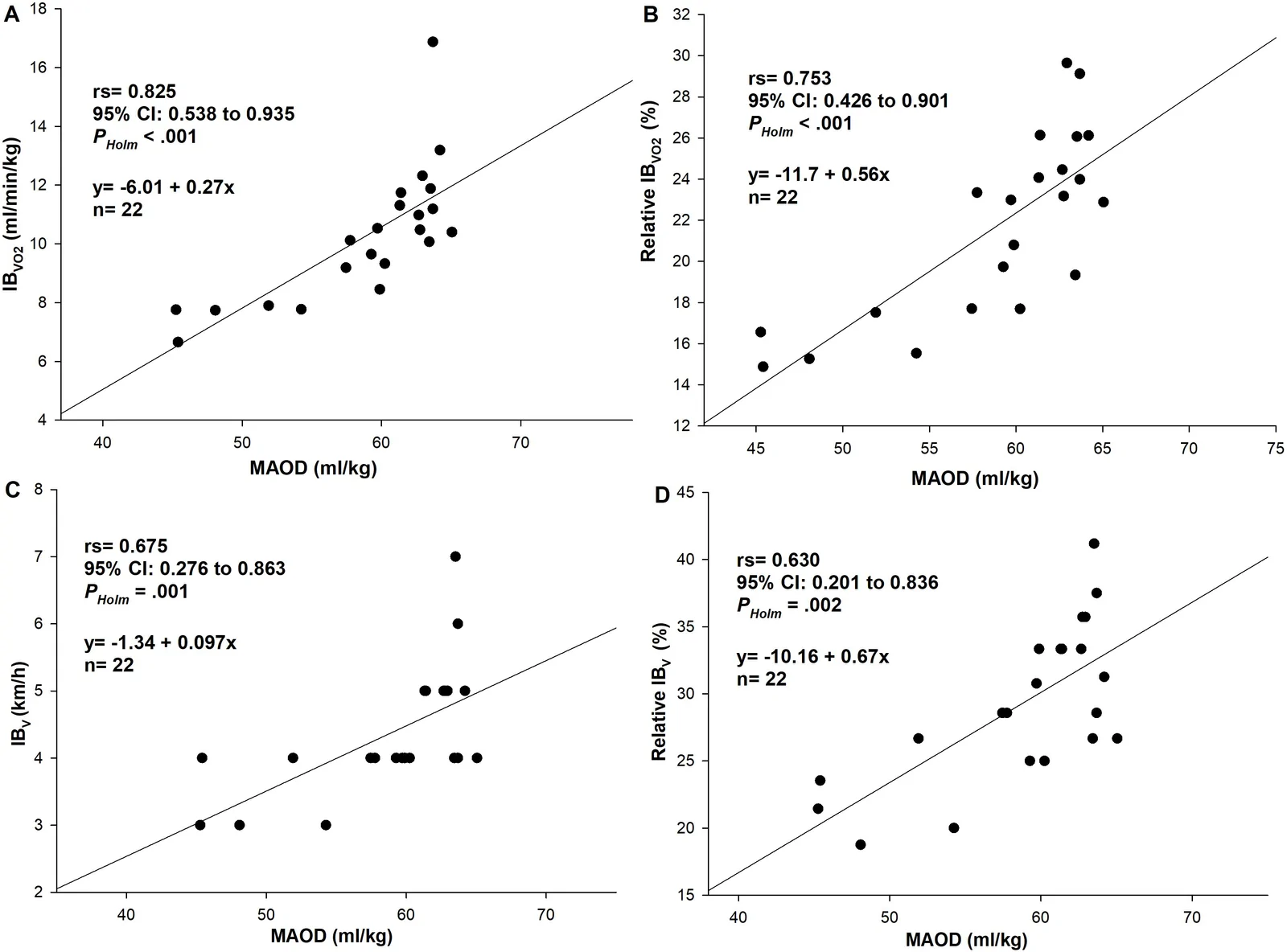
Relationships of maximal accumulated oxygen deficit (MAOD) with **(A)** absolute isocapnic buffering for oxygen (IB_VO2_), **(B)** relative IB_VO2_, **(C)** absolute IB for velocity (IB_V_), and **(D)** relative IBv. Associations were assessed using Spearman’s rank correlation coefficient (rs). Confidence intervals are bias-corrected and accelerated bootstrap 95% intervals based on 5,000 paired resamples. Holm-adjusted p values are shown.

In the exploratory analyses, peak power remained associated after FDR adjustment with absolute IB_VO2_ (r=0.701, q=0.003), relative IB_VO2_ (r=0.678, q=0.005), IBv (rs=0.517, q=0.047), and MAOD (rs=0.839, q<0.001), but not with relative IBv (r=0.475, raw p=0.026, q=0.081) (**Figures 3A–C**, **4A**). Mean power remained associated with absolute IB_VO2_ (r=0.590, q=0.017), relative IB_VO2_ (r=0.516, q=0.047), and MAOD (rs=0.665, q=0.005), but not with absolute or relative IBv (q>0.05) (**Figures 3B–D**, **4B**). The nominal associations of fatigue index with IBv (rs=0.452, raw p=0.035, q=0.105), relative IBv (r=0.444, raw p=0.039, q=0.113), and MAOD (rs=0.425, raw p=0.049, q=0.128) did not remain statistically significant after FDR adjustment. No HHV-related correlation remained statistically significant (all q>0.05).

**Figure 3:**
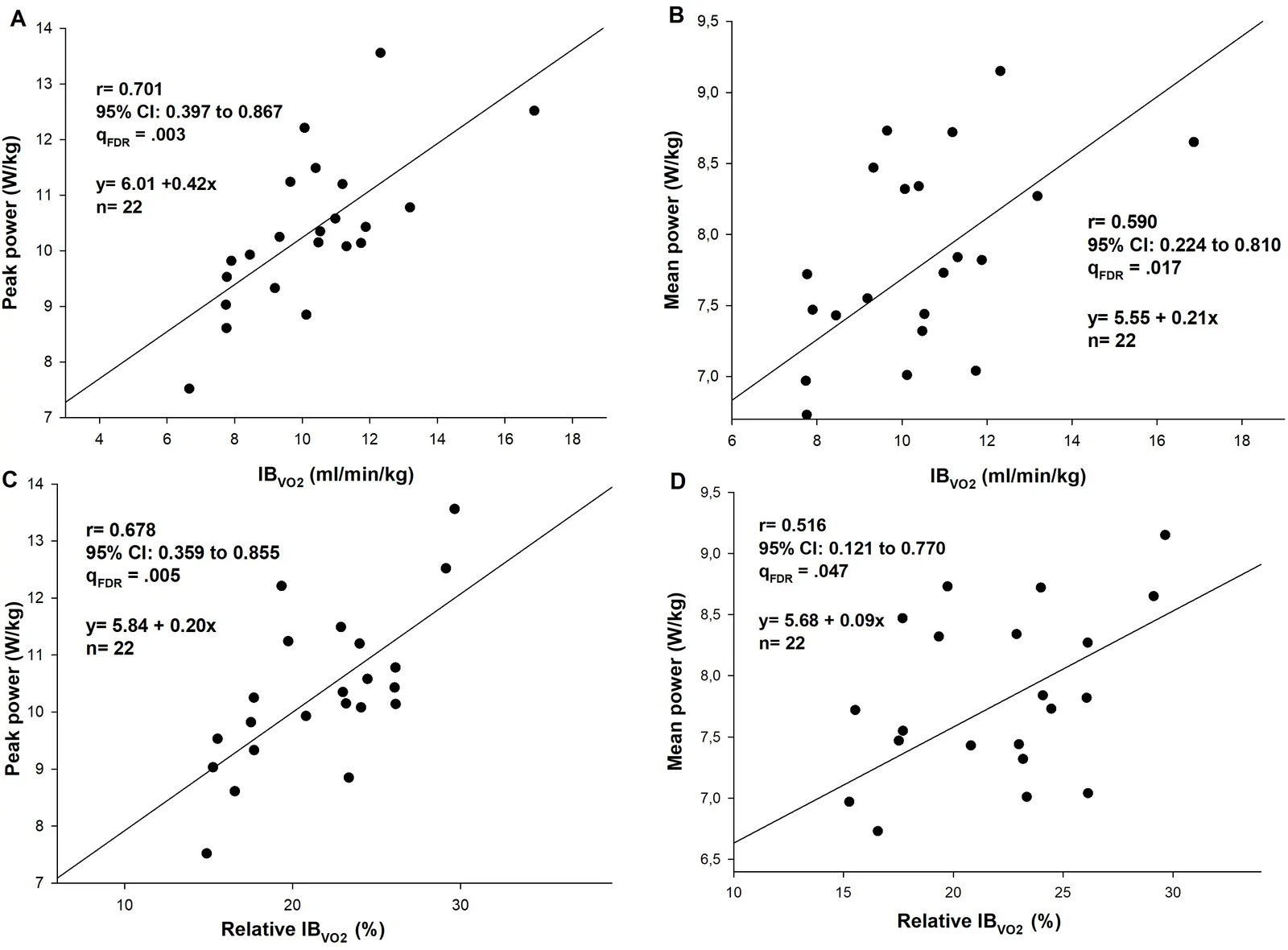
Relationships of **(A)** absolute isocapnic buffering for oxygen (IB_VO2_) with peak power, **(B)** absolute IB_VO2_ with mean power, **(C)** relative IB_VO2_ with peak power, and **(D)** relative IB_VO2_ with mean power. These relationships were assessed using Pearson’s correlation coefficient (r); fitted lines represent least-squares linear trends. Benjamini-Hochberg FDR-adjusted q values are shown.

**Figure 4:**
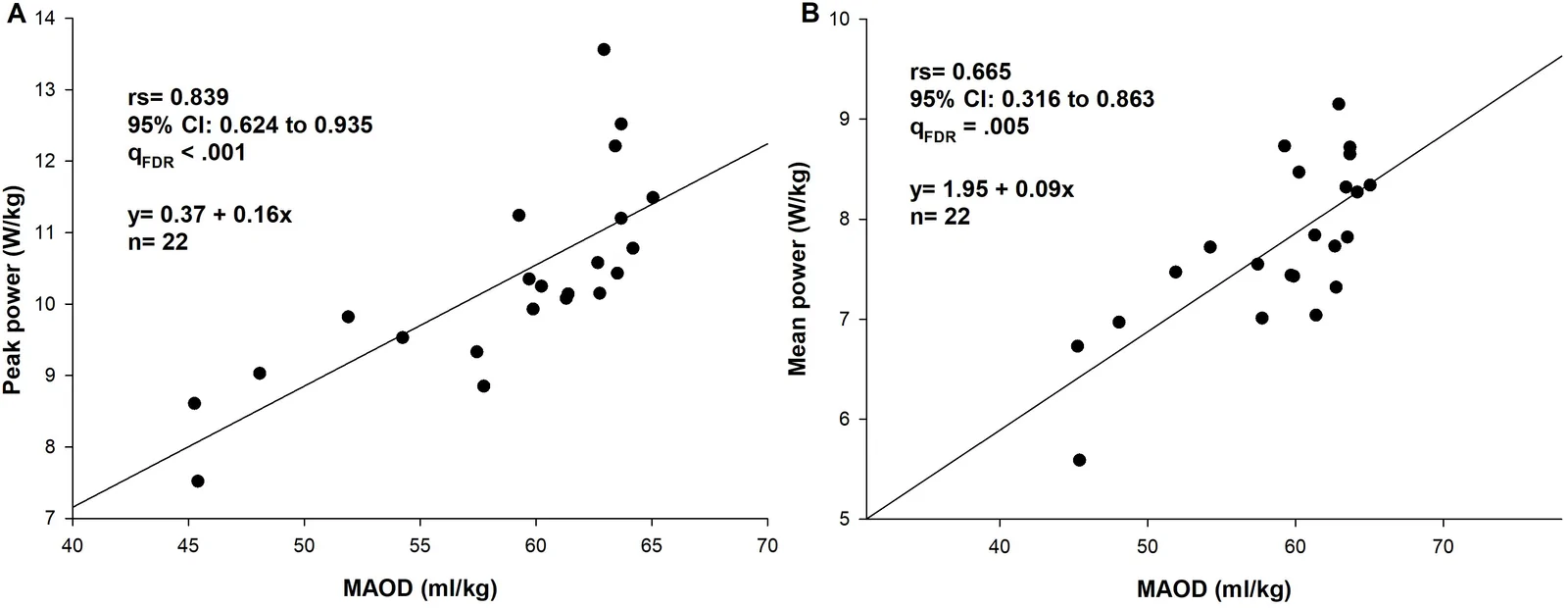
Relationships of maximal accumulated oxygen deficit (MAOD) with **(A)** peak power and **(B)** mean power. Associations were assessed using Spearman’s rank correlation coefficient (rs). Confidence intervals are bias-corrected and accelerated bootstrap 95% intervals based on 5,000 paired resamples. Benjamini-Hochberg FDR-adjusted q values are shown.

In the exploratory analyses, no statistically significant associations were detected between V̇O_2max_ or time to exhaustion and MAOD or any IB values after FDR adjustment (all q>0.05). AT_VO2_% was inversely associated with MAOD, absolute and relative IB_VO2_, IB_v_, and relative IBv (all q ≤ 0.017). ATv% was also inversely associated with all five variables (all q ≤ 0.044). RCP_VO2_% was not associated with MAOD or the IB values (all q>0.05). RCPv% was positively associated only with IBv (rs=0.590, q=0.017) (**Table 2**).

**Table 2:** Correlation coefficients with unadjusted p values and Benjamini-Hochberg FDR-adjusted q values for relationships of the maximal accumulated oxygen deficit (MAOD), the isocapnic buffering (IB) phase values with incremental exercise-test variables.

n= 22	MAOD (rs)	IB_VO2_ (r)	Relative IB_VO2_ (r)	IB_V_ (rs)	Relative IB_V_ (r)
V̇O_2max_	0.19 (0.396; 0.579)	0.24 (0.273; 0.450)	-0.13 (0.553; 0.670)	-0.17 (0.450; 0.602)	-0.24 (0.288; 0.450)
Time to ex	0.02 (0.934; 0.972)	-0.10 (0.657; 0.761)	-0.29 (0.194; 0.364)	-0.07 (0.760; 0.850)	-0.33 (0.131; 0.276)
AT_VO2%_	**-0.73** **(<0.001; 0.002)**	**-0.64** **(0.001;0.009)**	**-0.84** **(<0.001; <0.001)**	**-0.60** **(0.003; 0.017)**	**-0.69** **(<0.001; 0.004)**
RCP_VO2%_	-0.01 (0.954;.972)	0.16 (0.478;.623)	-0.07 (0.768; 0.850)	0.19 (0.406; 0.581)	-0.02 (0.935; 0.972)
AT_V_ %	**-0.53** **(0.011; 0.044)**	**-0.55** **(0.008; 0.031)**	**-0.68** **(<0.001; 0.005)**	**-0.60** **(0.003; 0.017)**	**-0.80** **(<0.001; <0.001)**
RCP_V_ %	0.24 (0.291; 0.450)	0.33 (0.132; 0.276)	0.32 (0.143; 0.290)	**0.59** **(0.004; 0.017)**	0.36 (0.103; 0.242)

Cells show the correlation coefficient followed by (unadjusted p; FDR-adjusted q). Pearson’s r was used when both variables were normally distributed; Spearman’s rs was used when either variable was non-normally distributed. Bold coefficients indicate q<0.05. V̇O_2max_, maximal oxygen uptake; Time to ex, Time to exhaustion; vV̇O2max, minimum speed which elicits V̇O2max; AT, anaerobic threshold; RCP, respiratory compensation point; ATVO2%, V̇O2 at the AT as % of V̇O2max; ATV %, running velocity at the AT as % of vV̇O2max; RCPVO2%, V̇O2 at the RCP as % of V̇O2max; RCPV %, running velocity at the RCP as % of vV̇O_2max_.

Relative AT was the only variable that showed a significant correlation with both MAOD and IB phases. Therefore, exploratory partial Spearman correlations were used to examine whether the association between MAOD and the IB phase persisted after controlling for the relative onset of AT. After controlling for AT_VO2_%, MAOD remained associated with absolute IB_VO2_ (partial rs=0.619, raw p=0.003, q=0.016). Associations with relative IB_VO2_ (partial rs=0.434, raw p=0.049, q=0.128), IBv (partial rs=0.439, raw p=0.047, q=0.128), and relative IBv (partial rs=0.262, raw p=0.250, q=0.435) were not statistically significant after FDR adjustment. After controlling for ATv%, MAOD remained associated with absolute IB_VO2_ (partial rs=0.746, q=0.002), relative IB_VO2_ (partial rs=0.632, q=0.013), and IBv (partial rs=0.527, q=0.047), but not with relative IBv (partial rs=0.405, raw p=0.069, q=0.173).

In the *post hoc* regression sensitivity analyses, adjusted R² values ranged from 0.434 to 0.559 (all overall p ≤ 0.002). VIFs ranged from 1.07 to 3.39. Residual Shapiro-Wilk tests (p=0.540-0.884) and Breusch-Pagan tests (p=0.066-0.144) did not provide evidence of major residual non-normality or heteroscedasticity. With HC3 standard errors, the IB coefficients were positive but imprecise for absolute IB_VO2_ (B = 1.866, 95% CI: -0.668 to 4.401; raw p=0.140, q=0.140) and absolute IBv (B = 2.931, 95% CI: -0.216 to 6.077; raw p=0.066, q=0.088). The coefficients for relative IB_VO2_ (B = 1.106, 95% CI: 0.341-1.870; raw p=0.007, q=0.028) and relative IBv (B = 0.715, 95% CI: 0.142-1.288; raw p=0.017, q=0.034) remained statistically significant after FDR adjustment. AT coefficients were not statistically significant in these models (all HC3 p≥0.116). Influence diagnostics identified observations above conventional Cook’s-distance thresholds, particularly in the absolute oxygen-uptake model (maximum Cook’s D = 3.11). Leave-one-out analyses preserved the positive direction of every IB coefficient, although nominal significance was not retained in every velocity-based refit. These analyses therefore do not establish that the IB-MAOD relationship is independent of AT.

## Discussion

The main finding of this study was that all four primary values of the IB phase were positively associated with MAOD, and these associations remained statistically significant after Holm adjustment. Exploratory analyses also identified several FDR-adjusted associations with Wingate peak and mean power. No statistically significant association was detected between V̇O_2max_ and either MAOD or IB phase values, suggesting that aerobic capacity may not be sufficient for interpreting the IB phase or anaerobic capacity in the present sample. These findings suggest that the IB phase may provide useful information related to anaerobic capacity; however, the small, heterogeneous sample and correlational design preclude causal or predictive conclusions.

The athlete may maintain performance during the IB phase without acidosis-related physiological limitation. During exercise above AT, the relatively high aerobic metabolic rate may expand the IB phase due to a slower rise in lactic acidosis (Yen et al., 2018). The ratio of aerobic to anaerobic metabolism in energy production during the IB phase may vary across different sports. Anaerobic-trained athletes, with higher skeletal muscle glycolytic capacity, have the ability to compensate for metabolic acidosis by improving buffering capacity (Hasanli et al., 2015; Edge et al., 2006; Bishop et al., 2008; Sharp et al., 1986). This type of metabolic adaptation increases the contribution of anaerobic metabolism during high-intensity exercise and improves athletic performance. The positive correlation between IB and MAOD, together with Wingate test data, may explain this relationship. The IB phase may be used as a physiological indicator of anaerobic capacity, as shown by Hasanli et al. (2015).

In this study, we showed a strong correlation between the IB phase, assessed as both oxygen uptake and running velocity, and MAOD. Our finding is consistent with the results of Hasanli et al. (2015), who found a significant positive correlation between the relative IB, as calculated from blood lactate levels, and MAOD (r = 0.73). Furthermore, anaerobic-trained athletes exhibited greater relative changes in blood lactate during the IB phase and higher MAOD than aerobic-trained athletes (Hasanli et al., 2015). Many studies have shown that anaerobic-trained athletes have higher IB phase (Röcker et al., 1994; Hirakoba and Yunoki, 2002; Korkmaz Eryılmaz et al., 2018), muscle buffer capacity (Edge et al., 2006), and MAOD values (Gastin and Lawson, 1994; Medbø and Burgers, 1990; Scott et al., 1991) compared to aerobic-trained athletes. Our IB phase and MAOD values align with previously published data (Lenti et al., 2011; Chicharro et al., 2000; Korkmaz Eryılmaz et al., 2018, 2024; Röcker et al., 1994; Zagatto et al., 2017; Gastin and Lawson, 1994; Medbo et al., 1988; Tanji et al., 2017).

In anaerobic sports, a higher proportion of fast-twitch muscle fibers is advantageous for achieving high performance. While it is not always possible to increase the percentage of fast-twitch fibers through anaerobic training, anaerobic capacity may be improved by increasing muscle buffering capacity (Scott et al., 1991; Edge et al., 2006; Bishop et al., 2008; Sharp et al., 1986). The increased buffering capacity may explain the enhancing effect of anaerobic training on the IB phase (Erylmaz et al., 2018) and MAOD (Tabata et al., 1996; Medbø and Burgers, 1990; Hov et al., 2023). In contrast, aerobic endurance training improves V̇O_2max_ without changing the IB phase (Chicharro et al., 2000) or MAOD (Tabata et al., 1996), nor does it change muscle buffering capacity (Sharp et al., 1986). Most of our sample consisted of team sport athletes, except for four sprinters. Team sports competitions and training typically involve intermittent anaerobic exercises. High lactate and H^+^ accumulation during high-intensity periods of intermittent exercise in team sports may improve buffering capacity by stimulating the adaptation of muscle pH regulatory systems (Edge et al., 2006). In this context, having a higher buffering capacity may provide an advantage for higher MAOD (Tabata et al., 1996), and this is correlated with our findings. The lack of blood lactate measurements is a limitation of our study. Since we do not have any data on blood lactate and pH, any assumptions will be speculative. Further studies are needed to clarify the relationship between the IB phase and MAOD by directly measuring both muscle and blood buffering capacity, as well as the two variables mentioned.

A significant negative correlation was observed between relative AT (as a percentage of V̇O_2max_) and both MAOD and the IB phase values. This may indicate that athletes who reach AT earlier may have higher anaerobic capacity with similar vV̇O_2max_. However, partial Spearman analyses revealed a correlation between MAOD and IB phase independent of AT. On the other hand, after FDR adjustment, the number of variables with statistically significant coefficients decreased. Influence and leave-one-out diagnostics also indicated some instability in this small sample. Because IB is mathematically derived from AT and RCP, AT, RCP, and IB cannot be entered simultaneously without perfect structural multicollinearity. Our findings provide limited evidence that AT position does not fully account for the relative-measure associations, but they do not show that the IB-MAOD relationship is independent of AT. As AT indicates the onset of metabolic acidosis, an increase in this threshold is thought to enhance maximal exercise capacity (Oshima et al., 1998). Indeed, a rightward shift of the AT to higher exercise intensities is a characteristic effect of endurance training (Chicharro et al., 2000; Hoogeveen, 2000). AT may occur at a higher percentage of V̇O_2max_ in aerobic-trained athletes compared to anaerobic-trained athletes (Korkmaz Eryılmaz et al., 2018; Green et al., 2014). Conversely, anaerobic training may enhance buffering capacity without altering AT, enabling a longer IB phase by raising RCP (Erylmaz et al., 2018). If both AT and RCP shift upwards at the same rate, the IB phase may remain unchanged (Chicharro et al., 2000; Nakahara et al., 2020). To comprehend the mechanisms underlying adaptations from different training methods, changes in AT and RCP should be assessed as relative values.

RCP refers to the failure of the body’s buffering systems (Meyer et al., 2004; Whipp et al., 1989), making it difficult to sustain exercise above the RCP during incremental exercise. The enhanced buffering capacity and/or reduced chemosensitivity of the carotid bodies to metabolic acidosis resulting from training may delay the onset of the hyperventilation response (Hirakoba and Yunoki, 2002; Miyachi and Katayama, 1999; Takano, 2000; Nakahara et al., 2020). A larger IB phase, relatively higher RCP values, and consequently a smaller HHV phase are generally considered key characteristics of trained athletes (Lenti et al., 2011; Chicharro et al., 2000; Erylmaz et al., 2018). On the other hand, there are controversial data about the relationship between the HHV phase and exercise capacity (Oshima et al., 1997, 1998; Bentley et al., 2005; Korkmaz Eryılmaz et al., 2018). Our findings indicated that HHV values did not correlate with MAOD, V̇O_2max_, or the Wingate test variables. Beyond the physiological mechanisms that regulate ventilatory response to exercise, psychological tolerance to fatigue, and motivational factors may determine the HHV phase.

The importance of the IB for aerobic exercise capacity remains poorly understood (Hirakoba and Yunoki, 2002). In our study, we found no significant association between V̇O_2max_ and IB values or MAOD; however, given the small sample and a sensitivity threshold of approximately |r|=0.55, smaller associations cannot be excluded. Our findings support Bentley et al. (2005), who showed that the IB phase was not associated with exercise performance requiring aerobic endurance. In contrast to our data, several investigators have reported a positive correlation between the IB and V̇O_2max_ in aerobic endurance athletes, older adults, and patients (Oshima et al., 1997, 1998; Lenti et al., 2011; Yen et al., 2018). Therefore, they considered the IB phase to be an indicator of endurance performance. On the other hand, studies with subject profiles consisting of both aerobic and anaerobic athletes have shown a negative correlation between the IB phase and V̇O_2max_ (Hasanli et al., 2015; Hirakoba and Yunoki, 2002; Korkmaz Eryılmaz et al., 2018). Based on our findings and those of previous studies (Hasanli et al., 2015; Röcker et al., 1994; Hirakoba and Yunoki, 2002; Korkmaz Eryılmaz et al., 2018), it can be suggested that the V̇O_2max_ may not be sufficient to interpret the IB phase. The relationship between the IB phase and aerobic or anaerobic capacity may differ among athletes, depending on metabolic pathways during training sessions and the resultant adaptive responses. The heterogeneity of our sample across sports disciplines and the small sample size, which may affect the precision of correlation coefficient estimates, are limitations of our study. Further studies are needed to clarify inconsistencies in this finding by accounting for participants’ training status (Lenti et al., 2011).

The maximum anaerobic energy produced per unit of time, known as anaerobic power, and the total energy that can be resynthesized by anaerobic metabolism during maximal exercise, referred to as anaerobic capacity (Heck et al., 2003), are the two key variables used to evaluate anaerobic profile in the Wingate Anaerobic Test (Inbar et al., 1996). The correlation between mean power and MAOD in our study indicates that both tests are consistent in assessing anaerobic capacity. Additionally, in line with previous research (Scott et al., 1991; Maxwell and Nimmo, 1996), the correlation between MAOD and peak power suggests that MAOD may also be related to anaerobic power. Furthermore, the FDR-adjusted associations between both peak and mean power and IB values may suggest that athletes with a larger IB have a greater anaerobic component in both power and capacity. However, these were secondary exploratory analyses and do not establish interchangeability, prediction, or a causal relationship between the Wingate test, MAOD, and IB phase measures.

## Conclusion

The presented study showed a relationship between the IB phase and anaerobic capacity, as assessed by both MAOD and the Wingate test. No significant association was found between V̇O_2max_ and either MAOD or IB phase values in the present sample, suggesting that V̇O_2max_ may not provide information related to the IB phase. These findings suggest that the IB phase may be a potential physiological indicator associated with anaerobic capacity in athletes, requiring further validation. A high buffering capacity to exercise-induced acidosis may allow anaerobic glycolysis to continue for a longer period, thereby providing greater anaerobic capacity. Monitoring the IB phase, MAOD, and V̇O_2max_ in athletes may allow coaches to adjust training programs to meet desired physiological demands.

## Data Availability

The raw data supporting the conclusions of this article will be made available by the authors, without undue reservation.
